# The meaning of culture in nursing at the end of life – an interview study with nurses in specialized palliative care

**DOI:** 10.1186/s12904-024-01493-5

**Published:** 2024-07-06

**Authors:** Rasha Mian, Åsa Rejnö

**Affiliations:** 1 Community Health and Medical Care, Northeast, Gothenburg, Sweden; 2 Department of Health Sciences, University West, Trollhättan, Sweden; 3 Skaraborg Institute of Research and Development, Skövde, Sweden; 4 Department of Medicine, Skaraborg Hospital, Skövde, Sweden

**Keywords:** Culture, End of life, Palliative care, Person-centred care, Specialized palliative care

## Abstract

**Background:**

The countries of the world are becoming increasingly multicultural and diverse, both as a result of growing migration, of people fleeing countries at war but also due to increased mobility related to labour immigration. Culture is a broad concept where the definitions focus on learned and shared values, traditions, and beliefs of a group of individuals. People’s culture affects health and perceptions of illness as well as treatment, symptoms, and care. Moreover, people who are at the end of life, live and exist within all levels and contexts of care. Specialized palliative care requires that the nurse has sufficient knowledge and skills to be responsible for meeting the patient’s nursing needs also on a cultural level, regardless of cultural affiliation. The aim of the study was to highlight nurses’ experiences of the meaning of culture when caring for patients at the end of life in specialized palliative care.

**Methods:**

The study was conducted with a qualitative design and inductive approach. Semi-structured interviews were conducted with twelve nurses in western Sweden. Data were analysed using qualitative content analysis.

**Results:**

The nurses had an awareness of culture as a phenomenon and how it affected palliative care at the end of life. The results showed two categories, *Awareness of the impact of culture on nursing* and *Culture’s impact and influence on the nurse’s mindset and approach*, consisting of seven subcategories that highlight the nurse’s experience. It emerged that there are differences between cultures regarding notions of dying and death, who should be informed, and treatments. There were also challenges and emotions that arose when cultural preferences differed among everyone involved. A person-centred approach allowed for recognition of the dying person’s culture, to meet diverse cultural needs and wishes.

**Conclusion:**

Providing culturally competent care is a major challenge. There are often no routines or methods prescribed for how nurses should relate to and handle the diversity of cultural notions that may differ from the values and cornerstones of palliative care. Having a person-centred approach as strategy can help to better manage the situation and provide equitable care on terms that respect cultural diversity.

**Supplementary Information:**

The online version contains supplementary material available at 10.1186/s12904-024-01493-5.

## Background

Cultural competence in nursing contributes to promoting health and well-being and creating the conditions for patients to face their illness, difficulties, and death in a dignified way based on their cultural background [1]. Many societies of the world are becoming increasingly multicultural as a result of growing migration both as an effect of people fleeing countries at war and due to increased mobility related to labour immigration, climate crises, poverty and global inequality. The increasing proportion of migrants globally – 240 million international migrants and additional people moving within their country of birth – leads to an increased diversity of cultures in many countries and thus an increase in patients with diverse cultures needing health care [2]. According to IOM UN Migration, every eighth nurse also works in a country other than where they were born or trained [3]. Care involving encounters between people with different cultures is a result of both perspectives of migration. Moreover, nurses can encounter people who are at the end of life in all levels and contexts of care. In this situation, they should be given good care based on their individual needs, regardless of cultural affiliation. People’s equal rights and opportunities regarding improved health (Goal 3 of the sustainable development goals) and reduced inequality (Goal 10) are important goals to reach, regardless of factors such as ethnicity, religion, and culture [4]. In the field of health care, one’s culture affects beliefs about both health and disease as well as treatment, symptoms, and care. Culture is a broad concept that is defined in a variety of ways, but most definitions focus on learned and shared values, traditions, and beliefs upheld by a group of individuals [5]. Despite any conceptual confusion, the core components of cultural competence models have been identified as cultural sensitivity, cultural knowledge, and cultural skills [6].

Every year, the WHO [7] estimates that 56.8 million people in the world are in need of palliative care. WHO defines palliative care as an approach which aims to improve the quality of life for patients and their families [7]. Palliative care should prevent and relieve suffering through early detection, careful analysis, and treatment of pain and other psychosocial, physical, and existential symptoms. The WHO definition has previously prevailed globally but has been complemented by an expanded consensus definition that the International Association for Hospice and Palliative Care (IAHPC) worked out and proposed, where pending death is not necessary but severe suffering is sufficient to be covered by palliative care [8]. This means that the consensus definition includes more persons than the previously prevailing definition. One of the points highlighted is that in order to provide good palliative care, it must be given with respect to the cultural values and beliefs of the patient and those close to them. Culture is important for the way patients react to symptoms, care, and facing death. When patients, families, and healthcare professionals come into contact with serious illness, difficult decisions, and limitations to treatment, cultural differences can become more prominent in the encounter [9].

For nursing in palliative care to be appropriate, considerations of cultural circumstances need to be taken into account [1, 10]. Cultural competence in care is a concept that has been developed to facilitate and enable care that can better respond to cultural diversity [11]. Cultural diversity is expressed in different ways in different countries and can vary and change as a result of migration, an aging population, and the fusion of different cultures [9]. According to Gysels et al., cultural differences and variations can also occur within different countries, regions, and groups. It is also important to distinguish between culture, ethnicity and religion. Although the concepts are closely related, equating culture with ethnicity does not capture the existing diversity in contemporary societies, and reducing it to personal beliefs has little explanatory value [9]. Culture is also influenced by gender, social class, education, age group etc. In addition, research from New Zealand describes variations in individual desires that also occur within and outside groups of people with the same cultural affiliation, i.e. sub-cultures [12]. For these reasons, it is important not to generalize and make assumptions based solely on cultural values but to inquire about the unique needs and desires that a patient has regarding end-of-life care.

Research has often stressed cultural knowledge as a way to gain cultural competence, but both cultural sensitivity (or humility) [13] and skills have also been shown to be of obvious importance [6, 10]. Emphasis is placed on the fact that nurses need to possess knowledge about other cultures to create understanding for their patients [1, 10], while nurses also need to be aware of their own cultural identity and how it affects their views on health, quality of life, and nursing.

Healthcare staff, patients, and relatives may have different values and approaches that do not correspond to the palliative care foundations and values regarding illness, treatment, and death [14]. This can concern different perceptions about life-threatening illness, communication, and decision-making about end-of-life care [15]. In Western culture, efforts are made to make it possible for the patient to be autonomous, thus making decisions about their care and the way they receive information about their health [12]. In Asian culture and Confucianism, the family’s involvement and role in care is important, and decision-making rests with them [16]. This can lead to conflicts in care when different cultural approaches, such as these cross paths at the end of life. One such area of conflict may be the differing perceptions regarding respect for patient autonomy, which is seen as important in Western culture, while the family is paramount in Asian culture [16].

Cultural aspects and care at the end of life have been studied to varying extents worldwide. The available studies highlight that knowledge of various cultural aspects is important in end-of-life care. Systematic reviews from both the United Kingdom [17] and Australia [18] show differences in care to the detriment of ethnic minorities, and these seem to persist over time. One common conclusion from these reviews is that persons belonging to ethnic minorities seem to be treated in a less person-centred way and more in line with healthcare professionals’ personal assumptions based on the patient’s cultural belonging. Differences between European countries are seen in areas such as decision-making processes in spiritual and religious matters, family presence, ethics, care, decision-making, autonomy, and the organization of care for the dying patient [9]. Although the staff may have training and experience in caring for people of varying origins, it is still experienced as challenging to care for and be aware of specific cultural factors and how these can affect different care needs at the end of life [19, 20]. These studies also support the requirement to be culturally competent and respect the patient’s preferences. Shortcomings and challenges resulted in neglecting the adaptation of care to the individual’s needs. Research from New Zealand describes how care staff handling the wishes of relatives’ belonging to ethnic minorities need to assume a different role in their patient’s care [12]. In addition to being experts in providing care, these caregivers take on a more educational and supportive role, providing support and equipping families with practical skills for training relatives to care for their dying elders. It has also been shown that the use of care teams and available resources specializing in palliative care is considered important for supporting staff and providing culturally specific care for patients and their families at the end of life [19].

Patients in need of palliative care are a growing group. When patients and persons important to them – hereafter referred to as “family members” regardless of family ties or not – encounter staff with different cultural backgrounds, various cultural preferences can emerge that are important to take into account. Despite the fact that there are many studies that shed light on the subject of culture and the end of life, it appears that care staff have a lack of knowledge and understanding regarding how to provide good end-of-life palliative care for people with diverse cultural backgrounds. The studies we found on the importance of culture in care have also invariably taken as a point of departure that the staff represent the culture of their own country, while the patient being cared for is the one who has ‘a different culture’ or belongs to an ethnic minority. This study intends to focus on the importance of culture in care by examining nurses’ encounters with patients from cultures other than their own in end-of-life care. This is regardless of whether it is the nurse or the patient who has ‘a different’ cultural background.

## Aim

The aim of the study was to highlight nurses’ experiences of the meaning of culture when caring for patients at the end of life in specialized palliative care.

## Method

The study had a qualitative descriptive design [21] and used individual interviews to highlight the nurses’ experiences [22]. The Standards for Reporting Qualitative Research (SRQR) checklist was used [23].

### Setting and participants

The study was conducted within care facilities that provide specialized palliative care in western Sweden in both inpatient palliative care units and care teams that provided specialized care in patients’ homes. For the study, strategic sampling and the snowball method were used in combination [21]. The inclusion criteria for participating in the study were that one had to be a nurse with at least two years of work experience and had to have worked in specialized palliative care. A total of twelve nurses participated in the study. All participants were women between 26 and 65 years of age, and they had between 2 and 32 years of professional experience as nurses. Four of the nurses had specialist training in palliative care, three in oncology care, one in intensive care, and four had basic nursing training. Two of those with a specialization in palliative care also had specialization in oncology care. Four of the participants came from palliative consultant teams, two from palliative resource teams, three from advanced health care in the home, one from hospice and two from the palliative care department at a hospital.

### Data collection

The head managers for a total of six care facilities for various forms of specialized palliative care in western Sweden gave permission to interview nurses within their operations during working hours. The managers forwarded the information letter about the study to nurses at their workplace. Five nurses who were interested in participating were recruited in this way. Through contact information found in the information letter, they contacted the first author to schedule an interview. To recruit additional nurses, the snowball method was used. Nurses from the same care organization that RM works in and from her network spread the information letter about the study and the possibility to participate. This resulted in seven nurses who were interested in participation and met the inclusion criteria. Contact was made with interested nurses to book the interviews. The interviews were conducted by RM during the period February-March 2022. Ten of the interviews were conducted digitally, and two were conducted at the nurses’ workplaces. They were conducted in secluded, undisturbed premises, which the participants themselves selected and during time points that were selected based on their activities. A semi-structured interview guide with questions based on literature in the field and the authors’ clinical experience was designed for present study [22]. The interview questions and interview procedure were tested on two of RM’s colleagues who were nurses but were not participating in the study, after which the questions were slightly revised based on their comments.

The interviews began with the same broad, open question ‘Can you tell me about your experience of the meaning of culture when caring for patients at the end of life?’ The question gave the participants the opportunity to speak freely about and define the concept of culture according to their own understanding, so as to describe their experiences in their own words. Follow-up and in-depth questions of the type ‘Can you tell me more about…?’ were asked. The interview guide was used to ensure that all questions were covered but not in any specific order. This was to be compliant with what the nurses told us while also keeping the thread of the interview on track. During the interviews, the interviewer made every attempt to be open to what the nurses told, thereby capturing as broad a description of the area as possible. The participating nurses’ own sense of what culture is was not questioned since it was their experience of the meaning of culture in care that was of interest. When the interviews with the nurses were conducted, the material was judged to have a rich variety and a depth that was sufficient for answering the aim. The interviews were recorded with a stand-alone digital tool, and after the interviews, they were transferred to secure storage in a password-protected computer. The recorded interviews were transcribed verbatim by the interviewer and lasted 314 min (mean 26 min). In all, they comprised 78 pages of transcribed text.

### Analysis

For the study, qualitative content analysis with an inductive approach, according to Graneheim and Lundman’s description [24], was used since this method is well suited to analyse the content of interview data. The analysis began with both authors reading through the interviews first as a whole and then each interview separately to get a grasp of the whole. Then, the processing of what the interviews told us began with RM extracting meaning units from the text related to the purpose of our study. The meaning units were then condensed and provided with codes that described their content, resulting in a total of over 400 codes. The codes were sorted by the authors into preliminary categories according to similarities and differences until consensus was reached. Some codes fitted into several categories, and this was resolved by the authors returning to the text of the interviews to be able to sort these codes into the category where they best belonged. Finally, the results came to consist of two categories, built up of a total of seven subcategories. For example, of the analysis process, see Table 1.

**Table 1:** Example of the analysis process with meaning units, condensation, codes, subcategories, and categories

Meaning units	Condensed meaning units	Codes	Subcategories	Categories
‘It’s sometimes hard to balance the demands we have on ourselves when it comes to information’. Now it’s like this you won’t survive this. And in some cultures they are not told to the patient, but to family members	Difficult to balance the demands we have for information. This is how you won’t survive this. In some cultures, you don’t tell the patient, but you tell the family members.	Information must be given to family members	Culture-specific beliefs about information and treatment	Awareness of the impact of culture on nursing
If it’s about the hope when they say what they want to do yes. You can’t take that away from them and say no, you’re not getting out of here, no. I wouldn’t say that regardless. You get to be in your own little bubble.	If it’s about their hopes when they say what they want to do. You can’t take that away from them and say no, you’re not getting out of here. I wouldn’t say that.	Don’t take away hope	Person-centred approach gives culture a voice	Culture’s impact and influence on the nurse’s mindset and approach

#### Ethical considerations

The study was carried out in accordance with the guidelines for empirical studies in Sweden. Permission to interview the nurses was given by the respective ward manager. No formal approval from the Regional Ethics Review Board was required according to The Act concerning the Ethical Review of Research Involving Humans: SFS 2003:460 § 3 [25] for this kind of non-interventional study involving healthcare professionals, which does not involve any risk of processing sensitive personal data; hence, no ethical review was made since the act does not apply to research not covered by the law.

Nevertheless, our work followed the ethical principles for medical research in the Declaration of Helsinki [26]. The nurses were given both oral and written information, and written informed consent was obtained. The voluntary nature of participation and the possibility of withdrawing without prejudice were explained. The identities of the nurses are protected by the confidential handling of data in accordance with EU regulations [27], and the results are described in such a way as to conceal the identity of any individual nurse.

The authors took into consideration the fact that the interviews could be experienced as stressful or that the nurses could take offense. It could be that during the interview, they would be reminded of situations that were perceived as challenging or where the patient was not perceived as having received good care. The interviewer was attentive to situations such as these arising and was prepared to give the participants the opportunity to take a break or discontinue participation if they themselves wished to do so, without having to provide any explanation. This situation did not arise during any of the interviews. The data are safely long-term stored and will be archived according to the Archives Act [28].

## Results

To give a common ground for understanding of the subject, the nurses described how they apprehended the concept culture, as people living their lives at different stages and in different family situations, with different gender identities and social life patterns. Furthermore, it was considered that culture can imply duty and responsibility as well as influence how people relate to situations around the person with an illness.

It also emerged that there were differences in care cultures between different care organizations, such as between hospice, hospitals, and home health care. There are for example differences between the ways the various care organizations view the number of visitors allowed to visit the person who was dying, at the same time. The nurses emphasized that culture, ethnicity, and religion are connected and influenced by each other but still separate entities. This meant that culture was not dependent on citizenship, but people within the same society, group, and municipality could have different cultures.

...The (difference in) culture can be greater between two people who grew up in the same municipality than between someone who comes from the Middle East and someone from South America... (Nurse 12)

There was an awareness among the nurses that culture as a phenomenon influenced and determined how everyone involved related to end-of-life care and nursing. The nurses stated that it was important to understand and take into account how culture can affect the care of the patient and their family members. They described culturally rooted traditions as playing a major role in the way the patient wanted to spend this last phase of life. The nurses stated that it was important to be aware of this so that they did not miss things that from a cultural perspective were considered important for the person being cared for.

### Main results

From the interviews with the nurses, two categories concerning their experiences of the meaning of culture when caring for patients at the end of life in specialized palliative care emerged, *Awareness of the impact of culture on nursing* and *Culture’s impact and influence on the nurse’s mindset and approach*, see Table 2. These were built up of seven subcategories which highlight the nurse’s experience of the importance of culture when caring for patients in specialized palliative care at the end of life. The results are presented through quotations under the respective category and subcategory.

**Table 2:** Results presented in categories and sub-categories

Categories	Sub-categories
Awareness of the impact of culture on nursing	Language as a barrier to meeting cultural needs
	Influence of culture on family roles
	Culture-specific beliefs about information and treatment
	Beliefs about dying and death based on cultural identity
Culture’s impact and influence on the nurse’s mindset and approach	Challenges and strategies brought about by culture
	Person-centred approach gives culture a voice
	Cultural diversity deepens the nurses’ perspective

### Awareness of the impact of culture on nursing

The nurses had an awareness of the impact of culture on nursing. They told about *Language as a barrier to meeting cultural needs* and also described the *Influence of culture on family roles* as an important factor in care. *Culture-specific beliefs about information and treatment* also had an impact on the nurses’ work as did *Beliefs about dying and death based on cultural identity.*

### Language as a barrier to meeting cultural needs

The nurses said that having a common language to communicate and transfer information in the encounter with patients and family members was a significant factor for understanding and gaining knowledge of the patients’ cultural identity and wishes. The nurses used interpreters to help with conversations if they did not speak the same language as the patient but there were occasions where access to an interpreter was lacking. The nurses said that on these occasions, they were unsure whether family members who were used for interpretation were influenced by their cultural beliefs and truly interpreted what the care organization and the nurse wanted them to convey. Other situations the nurses mentioned were when the patient, despite the use of an interpreter, could not express culturally specific wishes due to their condition, such as reduced level of consciousness, confusion, speech impairment, etc. This was considered a limitation, as the nurses thus had too little knowledge of the patient’s cultural needs and wishes regarding care at the end of life.

I had very little knowledge of his worries and his concerns or what he preferred and what he’s been like before his illness, and what he wants his end of life care to be like (Nurse12)

The nurses believed that limitations in language, affecting communication and understanding each other, made it more difficult to create a direct relationship with both patients and their family members. This could result in the patient’s and the family members’ cultural wishes not being taken into account to the same extent, as they would be for patients who could communicate directly with the nurses. This could result in family members not receiving information or not having had the opportunity to provide information on a par with the situation for those who could communicate directly with the nurses. The daily dialogue that the nurse had with family members and patients was limited in situations such as these.

### Influence of cultural family roles

According to the nurses, the family members had a major role and influenced how the caring and care for the dying patient was given. Such things concerned for example contributing with information about how the patient wanted care to be carried out if the patient could not give that information themselves due to their condition. Family members were seen as the means that provided the possibility to fulfil the patient’s wishes at the end of life. The nurses said that there were different roles played by family members in different cultures and that it could be important to be aware of this when caring for patients at the end of life. In some cultures, the man is considered the head of the family with a responsibility to be strong and not show signs of weakness, even though this person could be affected by illness himself. In other cultures, it was the eldest son or another family member who took on a role as spokesperson. The spokesperson’s role was to guide the family and start different processes regarding death that were part of the person’s culture. The nurses said that the spokesperson was an asset for both the family and the nurse. It was through this person who family members could gain more confidence in the care provided.

In different cultures […] there is usually someone who has the main responsibility in the family. […]. It can be the eldest sibling, the father, the mother or another relative. Then, I try to talk to […] the person who has the main responsibility for when I can justify that I do so […] it becomes easier and [in this way I can] get the rest of the family on board (Nurse 2)

The nurses collaborated with the family members as an aid in gaining understanding about routines, rituals, customs, and religious beliefs regarding death to be able to fulfil the patient’s culturally based wishes.

### Culture-specific beliefs about information and treatment

In different cultures, there were a number of different beliefs that affected patients and family members’ views and that could influence and have significance for the care that the nurse provided at the end of life. Nurses often encountered situations in which culture-specific notions about information received had to be handled. The nurses understood and were well aware that patients and family members could have different ways of relating to information about illness. Sometimes the patients themselves did not want to tell their family members about their illness. Others said that the information provided by the nurse should be given to the family members initially, and then the family would decide how much information the patient was allowed to receive. The nurses believed that family members had good intentions and wanted to protect the patient by withholding the truth. Other reasons could be that family members felt that there was no point in telling the patient, as it meant that the situation would worsen for the dying person. The nurses described not being able to inform the patient about their illness as a barrier that could affect the care of the patient. Although the nurses assumed that many fatally ill patients were aware of their situation, this was unspoken. It was still considered problematic when the nurse did not know the patient’s own wishes at the end of life. The nurses struggled between their own, the family members’, and the patient’s culturally specific beliefs about not providing information concerning the disease and the laws in Sweden regarding the right to receive information about one’s condition. Despite the fact that there were differences regarding the handing over of information, the nurse had to adapt according to how much information could be provided, based on the situation.

The family should be told first and decide what is appropriate for the patient to find out. And the cases where we have had consent or what shall I say. Where the patient has said it is ok [that the family is the one who gets the information]. Then it feels ok. (Nurse 6)

The nurses also mentioned that at end-of-life, pain-relieving drugs were something they provided daily in their work. In encounters with patients and their family members, they could face different cultural beliefs about morphine as pain relief. The perception was usually negative and led to patients and family members deciding that the patient should not receive morphine as pain relief, based on their cultural mindset.

in that culture morphine is considered a drug and as religious people and believers they do not want to accept the pain relief. In other cultures, morphine is associated with something given to hasten death. Therefore, patients and relatives do not want morphine to be given. (Nurse 2)

According to the nurses, different cultural views between family members, patients, and healthcare staff could exist on what benefits the patient, from the point of view of nursing in palliative care. They said that both patients and family members in some cultures wanted full life-sustaining measures and treatments right up to the end of life, mainly in areas when it came to nutrition and intravenous fluids. The nurses stated that it was because nutrition and food were seen as a symbol of life, hope and recovery, but it could also be linked to religious beliefs in some cultures.

### Beliefs about dying and death based on cultural identity

Other beliefs that the nurses noticed in their encounters with people from diverse cultural backgrounds had to do with the differing views on dying and death. God and religion had a great influence on how people at the end of life behaved and talked about death. Some considered it taboo to talk about death and illness, while others left all responsibility for their future to religion and a God who decides. Differences regarding views about death led to difficulties in encounters, and this affected nursing. When the nurses in their professional role tried to inform and have a discussion about the fact that the end was approaching, the patient and family members did sometimes not want to know this.

We want to talk about death, but it is not we who decide when death will happen, it is God then. Then there can be a bit of a clash in the conversations there. (Nurse 7)

Conceptions of dying and death were not always dependent on having differing cultural backgrounds. Differences in perceptions were also found in patients with the same cultural background as the nurse.

### Culture’s impact and influence on the nurses’ mindset and approach

Through analysis of the data, we showed that the nurses saw the *Challenges and strategies that culture brings about*. They clearly stated that a *Person-centred approach* gives culture a voice and told about how they used that strategy in care. They also said that *Cultural diversity deepens the nurses’ perspective* and described how they also learned from colleagues through talking about the experiences had by other members of the nursing team.

### Challenges and strategies brought about by culture

There were a number of challenges and cultural clashes that the nurse faced in the end-of-life conversations with people from diverse cultural backgrounds. The challenges arose when the nurses found themselves in situations that revolved around the care of the dying patient, which did not correspond to the palliative care approach and the nurses’ obligation to comply with the Sweden’s laws and regulations. It emerged that it was difficult for the nurse to respond to different emotions that arose in the dying patient, such as worry, pain, suffering, and not being able to provide a sense of security when different cultural beliefs came into conflict with each other.

… The last weeks of this person’s life […] she said why am I in more pain why am I worried. Why don’t the meds help? Help me, help me. She was very […] worried and then you weren’t allowed to say anything (about her dying) …(Nurse 10).

Sometimes situations arose where the patient did not want to be cared for by the opposite sex. This could be a challenge that depended on the composition of the nursing team. It could also be difficult when the nurses could not accommodate the patient’s cultural desires to avoid discriminating against the health care staff of the opposite sex who was scheduled to care for the patient.

Various challenges and cultural clashes in encounters in care that the nurses described gave rise to feelings of frustration, helplessness, hopelessness, and powerlessness. The nurses felt that limitations occurred when carrying out examinations and assessments because the doctor or nurse was of the opposite sex to the patient or the patient was fully clothed, and items of clothing could not be removed. This combined with having little knowledge about the patient resulted in them not being able to perform their tasks in the way they considered best and as they were required to do.

The nurses dealt with the challenges and emotions that arose through different strategies. Some sought support through reflection and venting their worries with the nursing team. Others became solution-oriented to make the best of the situation and reach a middle ground together. The nurses also received help from other people around the patient, such as doctors who had more authority than the nurses or the person who represented the family, to reach everyone involved.

### Person-centred approach gives culture a voice

The nurses believed that regardless of the patient’s cultural background and their family, it was essential that the point of departure always remained the dying person and their wishes, in order to meet their nursing needs at the end of life. The nurses emphasized that the patient was cared for and given good care regardless of their cultural background. They said that it was important not to assume that patients wished to be treated in a certain way, based on what the nurses knew about their culture in general, but rather to base care on how each individual patient expressed their wishes, given their unique situation. By being open-minded and starting from what was important to the person, things in the person’s culture they considered important was highlighted. The nurses adopted a person-centred approach to clarify what the person considered important to have in focus and in that way various culturally specific wishes could be fulfilled, including practical administrative issues.

That a paper death certificate is needed, because we send these digitally to the tax office and it is there, but the imam needs it on paper and in hand. Then, we have to go out quickly and find this certificate and ask the doctor or the secretary to print it out and put it in an envelope. We are not allowed to send it by post, so we must hand over the certificate itself. (Nurse 11)

The nurses wanted to do a good job and tried to accommodate cultural differences, showing consideration by respecting culturally informed wishes as far as possible and doing so based on the possibilities within healthcare. Respect could be made possible by listening and taking in the wishes of both patients and their family members. Respecting their choices and wishes in different situations at the end of life, even though the nurses, family members, and the patient did not always agree. The nurses emphasized that the patient’s will and wishes were primarily in focus in situations where there were different views among family members regarding the patient’s care. The nurses believed that respect for cultural needs can be shown by asking the right questions, so as not to step on anyone’s toes, and by letting the patient be involved in the decisions that are made. To provide good care, the nurses worked from a holistic perspective and by creating a trusting relationship. The nurses took the time to listen to the person’s life story, and family members’ views were also taken into account. The nurses stated that an established trust made it easier to get the patient and family members to be compliant and get everyone on board with the care provided. Through asking questions, the nurses found out about the patient’s life and worldview, spiritual beliefs, culturally specific wishes, and approach to different situations concerning the end of life.

### Cultural diversity deepens the nurse’s perspective

According to the nurses, the encounter with patients and family members from a different culture than their own, meant that they were enriched in terms of knowledge in several ways. The situations in which the nurses were involved led to recognition, knowledge, and better preparedness for similar situations in the future. In addition, the nurses described that their newly acquired knowledge of how culture can influence nursing and care, including rituals, customs, traditions, and ways of thinking about death and dying in the end-of-life phase within specialized palliative care, contributed to personal development. Here, family members could play an important role.

… It may be that their children have lived in Sweden and grown up in Sweden who […] come and say when this will happen, we will have to pay attention to this and this. Because they know both cultures in a different way” (Nurse 3).

This new knowledge and awareness resulted in the nurses becoming better caregivers both as individuals and as a team. They said that it is easy to get stuck in old routines and ways of thinking, and therefore, it was rewarding to learn about other people’s cultural beliefs differing from one’s own way of thinking. It emerged that the nurses were aware of and reflected upon the fact that everyone carries different prejudices with them. They tried to be open-minded and challenge their beliefs through an increased awareness of their own prejudices, trying to keep them at bay. They described that having an understanding of their own and the patient’s culture, as well as getting into the mind-set of others, were strategies used to avoid prejudice. Furthermore, the nurses described being influenced by diverse cultures can at the same time be a strength that leads to increased knowledge and helps them learn from each other’s cultures.

## Discussion

The focus of this study has been on the meaning of culture in healthcare. For this, interviews with nurses working in specialized palliative care settings were performed. In the interviews, close attention was given to the descriptions of their encounters with patients from cultures other than their own, in end-of-life care. The nurses were aware that culture influenced and affected end-of-life care. They had a broad definition of the meaning of culture and did not equate culture with ethnicity, religion or language, even though these are stated to be closely related [9].

### Awareness of the impact of culture on nursing

The culturally bound notions that came up were areas that the nurses paid attention to and that, according to them, could affect end-of-life care for all concerned. Different conceptions regarding the reception and transfer of information about illness were described as a difficulty for the nurses, such as when family members did not want the nurse to inform the patient about their illness. The nurses considered this to be contrary to various laws in Sweden regarding the patient’s right to participate in decisions concerning their own care [29] and to receive information about their health status to promote the patient’s self-determination [30]. It also went against the nurses’ preferred way of working, based on the palliative care ideology [31]. Similar findings are described in the study by Bellamy & Gott [12], highlighting the problem of different approaches to information transfer about illness.

Withholding information from the dying person was perceived as a challenge for the healthcare staff, as it contradicted Western culture’s view of the patient’s autonomy, where information about one’s own illness is an important part of enabling informed decisions [31]. The basis of palliative care in a Western approach can here in parts be seen to clash with approaches in other parts of the world as information to the patient about illness, and their situation, is not considered an important value in all parts of the world as for example in Asian countries [16]. A growing awareness of the importance of cultural diversity in palliative care exists, and the consensus-based definition of palliative care developed in a collaboration between 88 countries [8] can be seen as part of this.

### Culture’s impact and influence on the nurse’s mindset and approach

Person-centred care focuses on providing individually adapted care based on the person’s unique situation, where the patient and family members are involved. The goal is to make visible and meet the person’s social, psychological, existential and physical needs [31]. The nurses in this study strove to identify the meaning and importance of culture in nursing for both the patient and family members by paying attention to the expressions of the patient’s culture. The nurses adopted a person-centred approach where the starting point is to see the individual person and their family members and involve and adapt the care according to their needs and condition. Person-centredness as a theory serves as a basis and holistic approach to care, where cultural needs are also included [31]. One of the starting points in person-centred care in the palliative context is that the patient shares their life story [31]. This has the potential to make the importance of culture for the person visible.

To improve understanding and provide a better preparedness to work with cultural issues that may arise when patient and family preferences differ, education might be helpful. Several studies indicate that courses on how to provide culture-specific care at the end of life are requested by healthcare professionals [32–34]. Although challenges, hardships, and difficult emotions emerged in the present study, the nurses did not spontaneously describe a need for training or courses regarding culture-specific care. A possible reason could be that the nurses who participated in the study worked in specialized palliative care, which requires advanced knowledge and training in nursing and is characterized by frequent encounters with complex practical situations. It is also possible that if a direct question concerning the nurses’ need of education would have been posed, the nurses might have affirmed such needs. A systematic review of cultural competence interventions have shown that knowledge, attitudes/beliefs, skills, behaviour and confidence may improve from such interventions, independent of their length, duration and type of approach [35]. However, evidence that the interventions also had an impact on and improved health care outcomes such as patient satisfaction and patients trust was weak. Self-reflection and interprofessional collaboration have also been suggested as central to implement cross-cultural training in palliative care [33].More research seem to be needed focusing not only on knowledge gain in the health care professionals but also on the interventions possibilities to make a positive difference in care.

The study showed that the nurses who worked and met people at the end of life, with different cultural backgrounds than their own, made great efforts to meet cultural needs based on the wishes of patients and family members. Though we did not study their success in this and we only have their words to support this strive. One possible reason for their efforts might be that that they saw the limited time left as valuable to all involved in the face of impending death. Death touches us all, since we are all going to die eventually, regardless of background. In palliative care there arose challenges, varying approaches, and different beliefs when caring for people with diverse cultural backgrounds. The way the nurses relate to them can contribute to an understanding of the patients’ situations that can be the basis for providing a good, dignified death [7] on equitable terms [4].

The results showed that the nurses reflected upon their own prejudices and culture and the effects thereof on professional encounters. This is, according to Leininger [1], part of the mindset for developing cultural competence. To provide and accommodate culture-specific care for people with cultural backgrounds different from the nurse’s, it is required that the nurse possess cultural competence that contributes to the nurse’s professional and holistic caregiving [6]. The concept of cultural competence can be interpreted as difficult to define and not easy to apply clinically in nurses’ daily work. This is supported by findings from a scoping review which showed dissimilarities in definitions of cultural competence in the included studies [35]. People with diverse cultures and patients in need of palliative care are constantly increasing [2, 3, 7]. Therefore, this is an urgent topic for everyone working in palliative care today. Through shared reflection in the nursing team, knowledge and experience about what is considered significant at the end of life in different cultures can be obtained from each other, leading to a joint learning [36]. This has the potential to bring the nurse one step closer to providing equal, high-quality care for all patients. Taking cultural wishes into account is also one step towards the Sustainable Development Goals 3 and 10 set by the UN [4]. Further research is suggested on of the meaning of culture at the end of life in specialized palliative care from the patient’s own perspective where aspects such as agency and integrity might be specifically interesting to explore.

### Strengths and limitations

The study captures specialized palliative care nurses’ views of the importance of culture for palliative care at the end of life. The semi-structured interview provided the opportunity to cover all of the questions in all of the interviews. Even if the questions were not always asked in the same order, they were responsive to the participants’ narratives. Ten of the interviews took place digitally due to the COVID-19 pandemic. This can be seen as a strength since it made it possible to reach out to participants in a wider geographical area but also a weakness since it is more difficult for the interviewer to pay attention to the participant’s body language and reactions when interviews are not conducted face to face [22, 37]. There might also be a negative impact on the relationship when interviews are digitally performed. Such things as technical aspects and the feeling of distance can play a role here [37]. The researcher who performed the interviews were aware of this and strove to create an openness in the interviews. The participants who were interviewed digitally openly told about their experiences and shared both perceived difficulties and shortcomings, indicating a permissive climate. Also no technical problems occurred during any of the digitally performed interviews. All nurses chose the form of interview themselves and the nurses who were interviewed digitally possibly chose that form since they felt comfortable and familiar with that. A lot of meetings in their care practice were performed digitally during this period, due to the COVID-19 pandemic.

The choice of participants and the method of collection are also important elements in assessing the validity of the study [24]. Both the strategic selection and the snowball method ensured that participants who were genuinely interested in the topic were included. The participants in the study came from various organizations that conducted specialized palliative care and had extensive experience. Several were also specially trained in palliative care or other areas. The nurses who participated had varied cultural backgrounds; there were nurses with both Swedish and other cultural backgrounds. These qualifications meant that data of rich depth and breadth were obtained from participants who had experience with end-of-life care, where the nurse and patient have different cultural backgrounds. The impact of the participants’ interest in the topic is unclear but possibly gives ground for more developed reflections coming forth than participants with less interest within this topic would have provided. We did not ask the participants about their cultural background or how they defined their own cultural belonging, which can be seen as a limitation. This was a conscious decisions since we wanted to focus on the meeting between the nurse and a patient with differing cultural backgrounds. This can be seen as a strength since that the focus has clearly been on the encounter between the nurse and persons with different cultural backgrounds, regardless of whether it is the nurse or the patient who represents the country’s culture, in this case, the Swedish culture. This has provided a broader and more nuanced picture of the importance of culture for end-of-life care than is usually found in research.

The validity of the study depends on trust in the researchers interpretation of the data and the measures taken to demonstrate this [21]. The validity is strengthened by using the content analysis process according to Graneheim and Lundman [24] for the manifest content of the data, which is carefully described in the Methods section. In the results, quotations are used to provide the opportunity to assess the study’s validity and categorization. The first author carried out the initial analysis, and this was then discussed with the co-author for reflection regarding the choice of meaningful units, coding, and categorization. Disagreements were resolved through discussion until a consensus was reached, in accordance with descriptions by Polit and Beck [21]. The degree of objectivity in the text is described affirmatively by the author. The two authors’ different experiences in palliative care – RM from specialized outpatient palliative care in an area with high multicultural representation and ÅR experience in general palliative care in hospital – as well as our differing cultural backgrounds constitute a strength. This meant that our own pre-understandings were balanced by the other’s pre-understanding and not allowed to take over in ways that were not supported by data.

Estimation of the study’s transferability of the results to other groups and contexts is facilitated by an accurate and clear description of the context, selection of participants, data collection and analysis process, which we strived to provide. The participants in the study came from various care units, had varied long and short experience of specialized palliative care, and had a variety of cultural backgrounds. One limitation is that all of the participants were women, but unfortunately, this represents the gender distribution of nurses too well. Overall, we maintain that this contributes to the transferability of the results to other similar facilities that provide specialized palliative care, both in Sweden and internationally.

## Conclusion

Providing culturally competent palliative care is a major challenge. In the values and cornerstones of palliative care, there are seldom prescribed routines or methods for how the nurse should handle diverse cultural beliefs that may differ from the nurse’s own. To possess knowledge of the importance of culture in end-of-life care can lead to a reduction in generalizations and preconceived opinions. Instead, it can lead to increased awareness and contribute to knowledge and understanding on the part of the nurse who meets with and cares for the patient and their family members with a cultural background other than their own. These patients are already in a vulnerable situation when they are cared for at the end of life.

Having a person-centred approach as a strategy as the nurses in the present study did, can help to more easily manage the situation and provide care on equitable terms. The knowledge that encounters with people with different cultures brings about, leads to nurses being better equipped to provide improved palliative care, where cultural needs are also taken into account. With experience and new knowledge, the nurse becomes a more professional caregiver.

In summary, it can be difficult for nurses to have knowledge of all the different cultures they encounter since culture is not something that is permanent or unchanging but can vary and change as a result of migration, aging populations, and cultural blends. Being aware that culture is important and possessing sufficient knowledge of the way it can affect nursing in palliative care, helps the nurse meet the patient’s needs on a physical, psychological, relational, existential, spiritual, and cultural level. A person centred approach is suggested as a strategy to achieve this. This has the possibility to contribute to that good palliative care is provided based on recognition of and respect for the cultural values, convictions, and belief system of the patient and their family members. This ultimately ensures equitable care on terms that respect cultural diversity in accordance with the goals for sustainable development.

### Electronic supplementary material

Below is the link to the electronic supplementary material.

Supplementary Material 1

## Data Availability

The datasets for this study are not publicly available because of the risk that individual privacy could be compromised; however, the datasets are available from the corresponding author on reasonable request.
