# Supplementary material for: The meaning of culture in nursing at the end of life – an interview study with nurses in specialized palliative care
Source: BMC Palliat Care. 2024 Jul 6;23:166. doi: 10.1186/s12904-024-01493-5 (PMC11227218; doi:10.1186/s12904-024-01493-5)
Supplement: Supplementary file 1 — Supplementary Material 1 [file 12904_2024_1493_MOESM1_ESM.docx]

**Interview guide**

*The meaning of culture in nursing at the end of life -An interview study with nurses in specialized palliative care*

**Interview questions**

Can you tell us about your experience of the importance of culture when caring for end-of-life patients in specialized palliative care?

Can you describe a situation you have encountered based on your experience in nursing a patient with a different cultural background than your own?

Can you describe the opportunities/challenges you experience when meeting people who are at the end of life with a different cultural background than your own?
